# Controlled Access under Review: Improving the Governance of Genomic Data Access

**DOI:** 10.1371/journal.pbio.1002339

**Published:** 2015-12-31

**Authors:** Mahsa Shabani, Stephanie O. M. Dyke, Yann Joly, Pascal Borry

**Affiliations:** 1 Centre for Biomedical Ethics and Law, Department of Public Health and Primary Care, University of Leuven, Leuven, Belgium; 2 Centre of Genomics and Policy, Faculty of Medicine, McGill University, Montreal, Quebec, Canada

## Abstract

In parallel with massive genomic data production, data sharing practices have rapidly expanded over the last decade. To ensure authorized access to data, access review by data access committees (DACs) has been utilized as one potential solution. Here we discuss core elements to be integrated into the fabric of access review by both established and emerging DACs in order to foster fair, efficient, and responsible access to datasets. We particularly highlight the fact that the access review process could be adversely influenced by the potential conflicts of interest of data producers, particularly when they are directly involved in DACs management. Therefore, in structuring DACs and access procedures, possible data withholding by data producers should receive thorough attention.

## Abstract 2

This Perspective examines the potentially adverse influence of primary researchers on the openness of genomic data sharing and recommends changes to data access review procedures to mitigate this.

## Introduction

Over the last decade, sharing the data of publicly funded genomic studies is an issue that has received widespread attention in the scientific community. Funding organizations, research institutes and journals promote data sharing to ensure the optimal (re)use of data by researchers. Nevertheless, because of inherent privacy concerns in using genomic data and the importance of respecting the wishes of data subjects, controlled-access mechanisms have been adopted to allow researchers to use such data [1,2].

Data access committees (DACs) have been set up as a major instrument in reviewing access requests. The way DACs have been designed and function, however, differs significantly from one committee to another [3]. While some DACs are constructed at an institutional or consortia level, with higher formality and regular meetings, others are formed by small groups and rely on rather ad hoc meetings [4]. The two main central genomic databases, namely, the database of Genotypes and Phenotypes (dbGaP) and the European Genome-phenome Archive (EGA), also exhibit different approaches to DACs [5]. Limited available information on the access procedures of other controlled-access genomic databases [6,7] makes evaluation of their access procedures difficult, if not impossible.

Despite the differences, the ultimate goal of access review is to grant access to qualified users for a set of approved uses. Accordingly, assessing conformity of the proposed uses of data to the original consent forms research participants signed and examining the qualifications of the researchers requesting access seem to constitute the main tasks of an access review. Furthermore, access review specifies the duties and responsibilities of data users by means of instruments, such as data access agreements co-signed by data users’ institutions.

However, in situations in which the same researchers that have generated data also control further downstream use of the data, there is a conflict of interest, which might undermine data sharing. There are several reasons researchers might not be willing to share data they have produced, or at least, not for the time being. As Bobrow observes, “researchers who spend time, effort, and ingenuity to generate, process, and manage large research data sets expect to get appropriate credit” [8]. Data producers’ concerns have also been recognized in a number of international policy statements, such as The Fort Lauderdale Agreement (2003) and the Toronto Statement on Pre-publication Data Sharing (2009) [2,9]. In this article, we suggest a number of potential solutions to address this concern: objectivity of access review, fairness of terms and conditions in access to data, and transparency and accountability. We argue that these core elements should be incorporated into the process of access review by both established and emerging DACs in order to best serve the interests of the various parties involved.

## Objectivity of Access Review

Many DACs are mainly composed of the primary investigators or researchers who produced data. This allows the primary investigators to monitor the data they have generated. Primary researchers’ direct involvement is believed advantageous in order to ensure that any specific concerns expressed by research participants have been taken into account in the access review, particularly in dealing with sensitive data. Conceivably, primary researchers are trusted by research subjects in safeguarding their interests and respecting their wishes in the course of further data uses [10].

Despite the perceived advantages, objective decision-making in access review may be affected as a result of such direct involvement. Indeed, the DAC structure and composition should secure an objective access review, which is conducive to fair and responsible data access. This is in keeping with the ultimate purposes of data sharing, to optimize the use of genomic databases as community resources [9,11]. Researchers’ feeling of ownership to data they generated or expectation to control downstream uses of data have been reported in previous studies and could unduly influence data access review [12]. Currently, data sharing policies and guidelines do not explicitly address this issue, so data producers are left to determine on their own how to structure their DAC.

If a DAC is independent, this concern may be mitigated. For example, dbGaP’s DACs are composed of United States federal employees who conduct the access review without the involvement of the data producers. This model, however, entails a central infrastructure set up by the research institutes or funding organizations. At the moment, the prevalence of such central infrastructures is limited. It is therefore necessary that funding organizations, research institutes or relevant government bodies set up such infrastructure and steer access review. National data archives, such as the United Kingdom Data Archive or the Finnish Social Science Data Archive, or international archives, such as the Consortium of European Social Science Data Archives, could serve as potential models. Despite the similarities in general goals of data sharing in various disciplines, it is important to keep in mind that the nature of data, funding, and associated concerns and interests of the involved bodies in downstream uses may influence the structure of central infrastructures in different disciplines.

It is essential that central data access infrastructures avoid unnecessary administrative burdens on access review and potential delays in access. Indeed, reviewing a large number of access requests in a timely manner demands a significant and sustainable investment. This underscores the importance of seeking a balanced approach that fosters both efficiency and objectivity.

A two-tiered access committee might be an alternative solution. Whereas a DAC or compliance office could review access requests on a case-by-case basis, an independent steering committee might function in an oversight and/or advisory role. Steering committees can be formed in institutions or at a consortium level and could be responsible for oversight of the access review conducted by DACs that involve data producers. The relationship between steering committees and DACs, along with the scope of their discretion, should be clarified to avoid potential redundancies in access review and establish the optimal mechanisms of oversight. Establishing clear guidelines on access review will be also crucial. Steering committees would monitor the extent to which DACs adhere to such guidelines and root out arbitrary decision-making.

A steering committee can offer consultation in controversial cases or provide broader policy-level observations and guidelines. Also, should any complaint arise from access review, the case can be referred to the steering committee for further inquiry. With this two-tiered system, users will be provided with an opportunity to appeal against the DAC decisions if they wish. This resembles the idea of “appellate” institutional review board (IRB) which was put forward by Lantos as a transparent and publicly auditable body to issue decisions in controversial ethics cases [13]. In the absence of steering committees, an independent ombudsman could take on this role.

## Fairness in Terms and Conditions of Access to Data

Researchers’ concerns about receiving credit for their data producing and sharing efforts warrant the establishment of a number of relevant policies and terms and conditions of access. For example, researchers sharing data may be reluctant to provide others with access to it before they have had time to analyze it themselves and to publish the results of their analysis. Publication embargos or moratoriums, which prohibit secondary data users from publishing on shared data for a certain amount of time, were introduced into data sharing policies to address this [2,14], though it has been noted the mechanism may lack clarity and may sometimes prevent non-competing analyses [15]. Even after publication, data withholding has been documented amongst academic geneticists, indicating that researchers sharing data may be influenced by other conflicts of interest as well [16]. While some credit for researchers sharing data can be secured via the data access procedure as discussed below, and this may help to reduce their concerns, fair data sharing requirements on the part of data producers should be established through data sharing policy and not left to DACs to oversee.

Researchers sharing data may benefit from the new collaborations this facilitates. However, collaboration, and co-authorship in particular, should follow authorship guidelines, and sharing data should not automatically entail collaborative opportunities, though it may enhance these. Incentivizing data sharing remains important, as data sharing has yet to become the norm and there are many perceived barriers and disadvantages to sharing, from a researcher’s perspective. Reward should be provided in the form of acknowledgment and citation, where possible, of shared data resources [17,18]. This may be encouraged or enforced through the data access procedure, if stipulated in Data Access Agreements, for example. There is also some evidence that sharing the data that underlie research publications may lead to increased citation of the publication itself [19]. Nevertheless, the main routes to providing credit for data sharing in academia ought to be through standard institutional and funding mechanisms [15], rather than through DACs.

Some resource projects require that users contribute the data that they have generated thanks to the resource (e.g., UK Biobank [20]). Others simply request that research using the data be published. Obligations such as these are seen as an expression of reciprocity that could potentially be employed in other data sharing scenarios in order to demonstrate more broadly the benefits of data sharing.

## Transparency and Accountability

Only rarely do DACs provide public information about their assessment criteria or the number of data requests received (and how many were accepted or rejected). It could be argued that, just as respect for privacy and confidentiality is necessary in order to build trust and develop valuable relationships, a sufficient degree of transparency is necessary for maintaining that trust and these relationships. Transparency is also often seen as an important component of governance in health research because it is conducive to stakeholder accountability, including researchers [21]. This is particularly relevant in a field like genomic research, where soft norms and internal self-regulation still play a considerable role but need to evolve rapidly in order to keep pace with scientific and technological developments. In setting up Standard Operating Procedures (SOPs) for a DAC, transparency can also be used to reassure research participants regarding the integrity and security of the data sharing activities and to promote more harmonized processes that will facilitate international exchanges of data.

When setting up a DAC, it should be a given that the procedures and governance framework of the data access, including access and refusal criteria, number of applications, etc., are to be made available openly, and are to be presented in a way that is understandable to patients, research participants, and members of the research community [22]. In doing so, special attention should be paid to the proposed scope of the sharing and the applicable data protection framework (i.e., physical, administrative, and technological security measures). This information should also be updated at regular intervals to remain current. Furthermore, we would argue that DACs would also have much to gain by keeping a public log containing a minimum amount of key information on approved and declined data access requests, data security incidents, and the manner in which they were addressed by the DACs. This log could contain items such as the date of the incident, its general nature, the date and manner in which the incident was addressed, and any future implications for the data sharing process. For security reasons, it would be justifiable for databases to be given a reasonable delay period to make this type of information public. The significance of such public reporting and its role in fostering transparency has already been stressed by some stakeholders and addressed by some DACs [23–25].

## Conclusion

In principle, the structure of DACs should facilitate a comprehensive and fair access review. Current DACs often hinge on direct involvement of data producers, which triggers some concerns considering potential conflicts of interest. We believe such concerns could be addressed by complementary governance mechanisms adopted by the parties involved.

Funding organizations are best placed to play a key role in fulfilling fair, efficient, and responsible access review. There are, however, a number of roadblocks to the active engagement of funding organizations. There are limited examples at the moment of such extensive engagement by funding organizations across the world, due to limited infrastructure or under-investment in this area. Funders’ higher involvement in an efficient access review system should be stimulated, and current best practices seen in different fields should be considered as examples.

Funding organizations need to be assisted by other bodies, such as journals and institutions (e.g., universities, hospitals, or other research institutes) [2]. Journals are particularly well placed to maintain oversight of publication policies embedded in access arrangements. Research institutions also have a crucial role in providing organizational support for researchers so that they can adhere to data access and sharing mandates arranged by funding organizations.

Ultimately, however, the success of data sharing policies depends on introducing streamlined and harmonized approaches to data access across research and funding organizations. Internationally recognized standards and access arrangements will invigorate interoperability among data producing entities and improve the scalability of central data archives and of fair, transparent, and accountable processes of data sharing and access.

## Abbreviations

DAC: data access committee
dbGaP: database of Genotypes and Phenotypes
EGA: European Genome-phenome Archive
IRB: Institutional Review Board
SOPs: Standard Operating Procedures
